# Vaccine-Preventable Conditions: Disparities in Hospitalizations Affecting Rural Communities in the Southeast United States

**DOI:** 10.3390/ijerph22040466

**Published:** 2025-03-21

**Authors:** Etienne Pracht, Christina Eldredge, Divyani Tangudu, Richa Phuel, Athanasios Tsalatsanis

**Affiliations:** 1 College of Public Health, University of South Florida, Tampa, FL 33612, USA; rphuel@usf.edu; 2 School of Information, University of South Florida, Tampa, FL 33620, USA; celdredge2@usf.edu (C.E.); dtangudu@usf.edu (D.T.); 3 Morsani College Medicine, University of South Florida Health, Tampa, FL 33602, USA; atsalats@usf.edu

**Keywords:** vaccinations, immunizations, hospitalizations, healthcare disparities

## Abstract

Vaccinations are among the most effective means of preventing hospitalizations related to infections. Despite this, high hospitalization rates for vaccine-preventable diseases strain available healthcare resources and imply deficiencies in primary care. Barriers to vaccinations exist, such as the recent pandemic, vaccine hesitancy, misinformation, and access to care. This study analyzes hospitalization rates due to vaccine-preventable conditions and identifies factors contributing to an increase in these rates in the southeast United States. This study used data from four different data sources. The data covers four pre-pandemic years (2016 to 2019) and the pandemic period (2020 to 2022). The analysis categorized the numbers and rates of hospitalizations for conditions with an available preventative vaccine across three age groups: pre-school aged children, school-aged children, and adults. Comparisons between school- versus non-school-mandated vaccines and a focus on differences between rural versus urban communities, as well as demographic characteristics (i.e., gender, race, and ethnicity), are included. Chi-squared tests were used to assess differences in this descriptive part of the analysis. Linear multiple regression was used to examine the independent influence of geographic location while accounting for potential longitudinal trends and the dimensions of the SVI, including socioeconomic status, household composition, disability, minority status and language, and household type and transportation. The dataset included data from 22,797,826 inpatient episodes, including 32,358 for which the principal reason for hospitalization was a vaccine-preventable condition, not including COVID-19. The analysis shows a consistent pattern characterized by higher rates of hospitalization for counties classified as rural. The pattern holds for preschool age (*p* < 0.001), school age (*p* = 0.004), and adults (*p* = 0.009). The differences are statistically significant in the white population (*p* = 0.008); in pre-school children, school-age children, and adults (*p* < 0.001); in females (*p* = 0.08 in pre-school, and *p* = 0.013 in adults); and black adults (*p* = 0.02). The regression results confirmed the findings of the descriptive analysis, indicating significantly higher rates in rural communities. Finally, the regression analysis also showed significantly higher rates associated with greater social vulnerability. This study highlights gaps in vaccination opportunities. These gaps can be seen geographically and in terms of social vulnerability, affected by factors such as poverty, language barriers, household composition, and access to care. Hospitalizations due to immunizable diseases were found to be higher in rural areas, particularly among adults. Communities with a high SVI show a significant increase in hospitalization rates. Community-engaged vaccination outreach programs and state policies could improve vaccination rates, and therefore, public health in rural areas, reducing hospitalizations, and lowering infectious disease risks in these areas.

## 1. Introduction

According to the World Health Organization (WHO), the use of vaccines prevents 3.5–5 million deaths per year from viral and bacterial infections such as measles, diphtheria, pertussis, and influenza [1]. Furthermore, vaccinations are among the most effective means of preventing hospitalizations due to complications from infections [2,3,4,5,6]. Vaccinations play a key role in the management of chronic conditions, such as asthma, chronic obstructive pulmonary disease, and diabetes, as these conditions can increase the risk of hospitalization due to complications from infections [7,8,9,10]. In addition to preventing hospitalizations, vaccines, for example, those for COVID-19, have been shown to reduce severity of disease during hospitalization [11].

While the ability to avoid hospitalizations due to uncontrolled conditions is never absolute, “immunizable” conditions (i.e., conditions which may be preventable with the use of widely available vaccines) are a prime example of hospitalizations which are likely preventable, as vaccines are highly effective in preventing diseases and their subsequent complications that may require inpatient services [12]. Preventable hospitalizations are inpatient episodes which can be avoided through timely and effective primary or preventative care in the outpatient setting [13,14]. These hospitalizations often represent a failure in the continuum of care, where gaps in access to preventative measures lead to acute exacerbations requiring inpatient treatment.

From a health systems perspective, tracking inpatient episodes related to immunizable conditions is particularly informative to the healthcare community as related hospitalizations represent the clearest example of being avoidable [13]. The rates of avoidable hospitalizations serve as a valuable gauge of the appropriateness and adequacy of access to ambulatory care [15]. Consistently high rates of hospitalizations related to ambulatory care-sensitive conditions indicate deficiencies in primary care. This information aids decision makers in managing scarce valuable resources.

The aim of this paper is to study inpatient episodes related to conditions for which widely available vaccinations exist in a large state located in the southeast of the United States with a population of over 22 million people. These diseases include diphtheria, poliomyelitis, measles, mumps, rubella, chickenpox, haemophilus influenzae type b (Hib), pneumococcus, hepatitis B, tetanus, whooping cough, hepatitis A, human papillomavirus (HPV), meningococcal disease, rotavirus, respiratory syncytial virus (RSV), shingles, and COVID-19, for which vaccines exist and are universally accepted as safe and effective [16]. The paper studies three age groups (pre-school aged children, school-aged children, and adults), while distinguishing between school- versus non-school-mandated vaccines and focusing on differences between rural versus urban communities [17]. This paper adds to the literature by examining potential factors associated with preventable hospitalizations. In addition, the longitudinal nature of the analysis highlights the impact of the pandemic on rates of vaccinations and hospitalizations.

## 2. Data and Methods

### 2.1. Data Sources

The study analysis integrates four distinct data sources. Source one is inpatient episode data from 2016 to 2023, including International Classification of Diseases, 10th Revision, Clinical Modification (ICD-10-CM) diagnosis codes used to identify patients hospitalized for an “immunizable” condition, are from the Florida Agency for Health Care Administration (AHCA). The AHCA inpatient data also indicate patients’ demographic characteristics (age, race, Hispanic ethnicity, and gender) and their county of residence. These data are aggregated at the county level to calculate rates per 100,000 population. The other three data sources are as follows: the U.S. Census Current Population Survey, used to obtain county population by age group; the National Center for Health Statistics (NCHS) Urban–Rural Classification Scheme from the Center for Disease Control and Prevention (CDC) used to categorize counties as rural versus urban [18]; and the Center for Disease Control and Prevention/Agency for Toxic Substances and Disease Registry (CDC/ATSDR) Social Vulnerability Index (SVI), which measures a “community’s ability to prevent human suffering and financial loss in the event of a disaster” [19].

The data includes four pre-pandemic years (2016 to 2019), followed by the pandemic period (2020 to 2023). While the CDC declared an end to the public health emergency associated with the pandemic in May of 2023, we include it in the latter for purposes of our descriptive analysis, which treats COVID-19 separately. The ICD10CM codes used to identify patients admitted for an “immunizable condition” as the principal reason for the hospitalization are indicated below. For the purposes of this study, we defined “immunizable condition” as a condition for which there is a widespread accepted vaccine available for prevention. The unit of analysis is the rate of hospitalization per 1,000,000 population.

### 2.2. Statistical Analysis

Three dimensions play an important role in the analysis. First, the analysis divides immunizable conditions into two groups, indicating whether the associated vaccine is currently required for enrollment in the relevant state’s public school system. Conditions associated with public school-mandated vaccinations include diphtheria (A36), poliomyelitis (A30), measles (B05), mumps (B26), rubella (B06), chickenpox (B01), Hib (B96.3), pneumococcus (J13, J18.1), hepatitis B (B16), tetanus (A33–A35), and whooping cough (A37). In contrast, immunizable conditions not associated with a requirement for vaccination to enroll in public school include hepatitis A (B15), HPV (R87.810), meningococcal disease (A39), rotavirus (A08.0), RSV (B97.4), and shingles (B02). COVID-19 (U07.1) is omitted from the latter list but will be examined separately as associated vaccines were not available for at least part of the study period. The second dimension involves age. Three age brackets were selected to align groupings used by the U.S. Census to facilitate the calculation of rates. They are pre-school-aged children (0–4 years), school-aged children (5–19 years), and adults (age 20 and older). The third analysis dimension classifies counties as either urban or rural. Counties classified by the CDC as micropolitan, meaning they contain an urban cluster with 10,000 to 49,999 residents, or non-core, indicating fewer than 10,000 residents, were classified as rural. Counties classified as being a large/fringe metropolitan statistical area (MSA) with over one million residents, a medium metropolitan area (250,000–1 million residents), or a small metropolitan area (50,000–250,000 residents) were categorized in the analysis as urban.

The descriptive analysis includes a heat map with a three-color scale transitioning from green (no hospitalizations for the specific immunizable condition) to yellow (the midpoint calculated as the 50th percentile) to red (the highest observed rate of hospitalizations for an immunizable condition). The purpose of the heat map is to help visualize rates and direct readers to areas or conditions that matter the most.

### 2.3. Multiple Regression

In addition to the descriptive analysis described above, we employed linear multiple regression methods to incorporate the influence of potential longitudinal trends as well as social vulnerability. The latter was measured using the SVI, which incorporates socioeconomic status (employment levels, the degree of poverty, and health insurance status), household composition and disability (age distribution, persons with disability, and single-parent households), minority status and language (distribution of minority status and English language fluency), and household type and transportation (multiple vs. single-family and vehicle access) into a single index. While the original intent of the SVI was to measure a community’s ability to respond to a hazardous event, we use it here to examine the impact of these social conditions on rates of hospitalizations for immunizable illnesses. Our main focus is the overall rate of hospitalizations, but we ran the model separately by gender, race, Hispanic ethnicity, and age group in order to provide a more detailed picture of the influence of potential covariates.

## 3. Results

### 3.1. A–Rates of Individual Conditions

Table 1 shows a heat map of the number of hospitalizations associated with an immunizable condition listed as the principal reason for the episode. The map includes three types of conditions. The top group includes all conditions for which public school children must be immunized. The second group contains conditions for which immunizations are optional. The third group, at the bottom, shows the number of cases associated with COVID-19. The heat map colors were applied separately to each panel to maintain relativity within the classification. Each group is divided into three panels based on patient age. All values in the map are rates per 1,000,000 population.

The top left panel relates to pre-school-aged children and conditions associated with public school-mandated vaccines. The map shows there were no cases (green) of hospitalizations for six of the eleven conditions (diphtheria, poliomyelitis, rubella, Hib, hepatitis B, and tetanus). These data indicate the sporadic occurrences of measles and mumps while hospitalizations for chickenpox are consistent, with a noticeable decline at the height of the COVID-19 pandemic. Two conditions, pneumococcal and whooping cough, stand out. Pneumococcal showed a significant pre-pandemic spike in 2018 (150 cases or 131/1,000,000) and 2019 (319 or 280/1,000,000). Whooping cough hospitalizations for pre-school-aged children averaged around 45 (39/1,000,000), falling to zero in 2021, but climbing to 37 (33 per 1,000,000) in 2023.

The top-center panel shows the number of hospitalizations for school-age children. Similarly to pre-school age children, in most cases, hospitalizations did not occur for the majority of conditions. A single case, involving measles, occurred in 2023. Similarly, there was a single episode involving tetanus in 2020. Mumps was the principal reason for hospitalization for twenty cases pre-pandemic, but for only one during and one after the pandemic. Chickenpox occurred with more annual consistency, with the exception of 2021 when the number of related hospitalizations was zero. While the number of cases involving pneumococcal was smaller compared to the pre-school age group, a similar spike occurred in 2018. Finally, the number of episodes caused by whooping cough was typically in the low single digits for this age group.

Adult hospitalizations caused by an immunizable condition associated with a school-mandated vaccination are depicted in the top-right panel. With the exception of hepatitis B, all conditions caused at least one hospitalization in this age group during the study period. One to three hospitalizations for poliomyelitis and measles occurred in most years (yellow blocks) while mumps caused four to eleven cases per year. The number of annual hospitalizations for chickenpox ranged from 32 (1.9/1,000,000) in 2021 (pandemic year) to 78 (4.7/1,000,000) in 2018 (pre-pandemic). In contrast to children, tetanus is a consistent cause of a handful of cases per year in the adult population. The number of annual whooping cough-related hospitalizations is consistently higher than in the school-age group but lower compared to the pre-school age cohort. The most noticeable difference compared to younger age groups relates to pneumococca-related hospitalizations which reached nearly 7000 (414/1,000,000) in 2019. The data show higher risk levels related to hepatitis A and shingles for the adult population. Rates of hepatitis A ranged from 10.4/1,000,000 in 2023 to 126.1/1,000,000 in 2019. The rate of shingles-related hospitalization averages 63/1,000,000 during the study period. Finally, the rates of COVID-19-related hospitalization peaked in 2021 at 8180/1,000,000 in 2021. Post/Long-COVID-19 hospitalizations peaked in 2022 at 3.4/1,000,000.

### 3.2. B–Rates by Metropolitan Status

**Figure 1:**
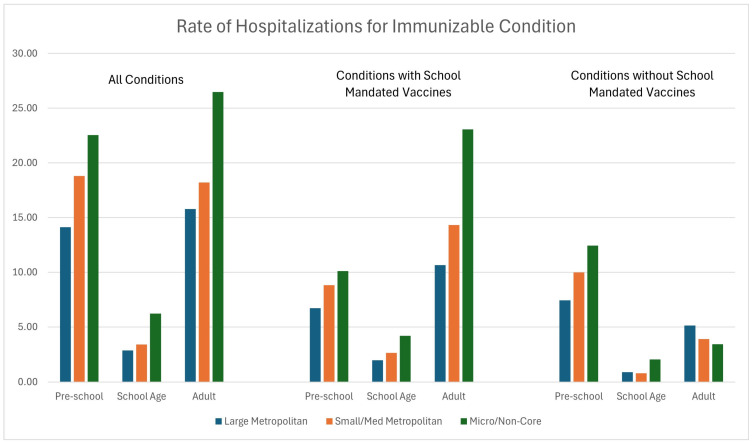
Rates of hospitalizations for immunizable conditions by metropolitan status.

Figure 1 shows the rates of hospitalization for an immunizable condition by metropolitan status. Rates were calculated for three definitions of immunizable conditions; either all combined or whether the condition is associated with a school vaccine mandate. The rates are further categorized by age group (pre-school age, school age, and adults). With the exception of non-school-mandated vaccines for adults, the pooled sample shows a consistent pattern characterized by higher rates of hospitalization for counties classified as more rural. The pattern holds for preschool age (*p* < 0.001), school age (*p* = 0.004), and adults (*p* = 0.009). The center set of bars in Figure 1 show the rates of hospitalizations for conditions associated with a school-mandated vaccine. While all three age groups show a similar rising pattern as counties become more rural, the difference is statistically significant only for school age children (*p* = 0.02) and adults (*p* = 0.002). It is not statistically significant for pre-school age children (*p* = 0.31). Finally, for conditions not associated with a school-mandated vaccine, the difference is not statistically significant for pre-school children (*p* = 0.32) or adults (*p* = 0.08). In contrast, the difference remains statistically significant for school age children (*p* = 0.019).

Table 2 contains the rates of hospitalization for the three categories of conditions (all, school-mandated, and not school-mandated). The data in the second column duplicate the information in Figure 1. The remaining cells show the patterns by race, Hispanic ethnicity, and gender. While the patterns indicate increased rates of hospitalization as counties become increasingly rural, the differences in most of the smaller sub-groups were not statistically significant, likely due to reduced power.

### 3.3. C–Social Vulnerability, Socioeconomics, Rurality, and Longitudinal Trend

Table 3 shows the results of the linear multiple regression models explaining the county level rate of hospitalizations for an immunizable condition. The rates used in this analysis, as before, did not include hospitalizations for COVID-19. The regression results confirmed the finding of the descriptive analysis, indicating consistently higher rates of hospitalizations associated with rural (noncore) areas. This result was consistent across all age groups. However, it did not hold in the models for non-white/black race or Hispanic ethnicity. The results also show higher overall hospitalization rates across the three age groups in counties with higher vulnerability indexes. Finally, the results did not show a significant longitudinal trend.

### 3.4. D–COVID-19

Table 4 shows the rates of inpatient episodes with COVID-19 as the principal reason for the hospitalization per 1,000,000 population. The rates are reported by metropolitan status (columns 2, 3, and 4) and race, Hispanic ethnicity, and gender (rows). The table is divided into three panels showing the rates for pre-school-aged children (top), school-aged children (center), and adults (bottom). With two exceptions, namely school-aged and adult females, rural areas predominantly experienced statistically significant higher rates of hospitalizations.

## 4. Discussion

In the literature, the importance of vaccines in preventing disease and complications has been noted. Further, previous studies on vaccine-preventable diseases in adults have shown significant increases in complications and worse outcomes [20,21]. Therefore, the results of this study on hospitalization rates for vaccine-preventative conditions in the southeast United States demonstrate opportunities to prevent disease by addressing gaps in vaccinations. Most notability, statistically significant hospitalizations related to conditions for which there are widely available vaccines were observed in rural areas, especially in adult populations. The rural/urban pattern was reinforced by the study results associated with COVID-19, the most recent condition to affect the population. Further, adults specifically were observed to have a statistically significant increase in hospitalization rates if living in a community identified to have a high SVI. The SVI encompasses four themes, socioeconomic factors (poverty, unemployment, lower education level), housing/transportation factors, household composition, and minority status/language factors, which adversely affect communities with inadequate access to, for example, healthcare resources. This analysis focused on overall vulnerability, indirectly highlighting the importance of access to primary care resources. We did not examine the role of the individual themes as that fell outside the scope.

The geospatial implications of the study’s findings are especially concerning in the southeastern United States where hurricanes and tropical storms are prevalent, several of which occurred during the study period. Further, this area is especially vulnerable to the increasing effects of climate instability. Gaps in vaccination rates may leave these areas particularly vulnerable to disease outbreaks in the event of a natural disaster when utilities (water, electricity) and access to healthcare are limited during these events [22].

Other notable findings of this study include the rebound of chickenpox and whooping cough hospitalizations in pre-school-aged children to or near to pre-pandemic levels. Interestingly, as of 2023, pneumococcal-related hospitalizations have not yet rebounded to pre-pandemic levels. Possible reasons for this include more remote work, less use of daycare facilities, and perhaps that fact that this condition is spread through direct contact and may not be as contagious as varicella or measles [23,24]. Additionally, some communities in the years 2022–2023 may have continued with infection precautions and social distancing post-pandemic.

Further, the data support the efficacy of school-mandated childhood vaccinations to protect against pediatric hospitalizations given the relatively low numbers noted on the heat map in the school-aged category. In contrast, the higher numbers of adult hospitalizations related to infections for which schools mandate vaccinations, in comparison to pre-school- and school-aged children, may be explained by waning immunity in adults. In other words, adults that were vaccinated as children may require boosters to maintain immunity to these diseases as they age.

Previous studies and reports have supported the efficacy of vaccines in preventing hospitalizations. The CDC’s Morbidity and Mortality Weekly Report (MMVR) states that childhood vaccinations among children born from 1994 to 2023 prevented 32 million hospitalizations [25]. However, despite the proven efficacy of vaccinations in reducing the burden of immunizable conditions and preventable hospitalizations, several barriers still exist to the immunization process for public health including the recent pandemic, vaccine hesitancy, misinformation, disinformation, and access to care. Increasingly, the literature notes that individuals exposed to misinformation (the conveyor is unaware of the falsehood) and disinformation (the conveyor purposely spreads falsehoods) may be hesitant to receive vaccinations due to safety concerns or distrust in the healthcare system [26,27,28,29]. Access to care, especially in low-resource, low-income [30,31,32,33], and rural [34] communities remains challenging with limited vaccination center availability [35], increased cost of vaccines [36], lack of public transportation [37,38], and waiting times contributing to gaps in immunizations in these populations.

To address these barriers, several approaches could be utilized. One approach could be investing in vaccine outreach programs for rural communities. Previous studies have shown success in using community-engaged approaches to vaccine program outreach in rural communities. Interventions have included “pop-up” and mobile clinics [39] and the use of short videos to improve health literacy regarding vaccines [40]. However, most of the previous literature focuses on COVID-19 education and vaccinations. Further research could address the need for adult boosters for typical childhood diseases, perhaps using similar study methods implemented in the recent pandemic to promote COVID-19 vaccination, such as increasing access through interventions such as mobile clinics, community partnerships and capacity building, “vaccine champions”, and social marketing campaigns [41,42].

Another strategy could be reducing gaps in childhood vaccinations for highly contagious diseases. By increasing vaccination rates among children, adult hospitalizations for these conditions may be reduced via the maintenance of herd immunity, especially when state policies support routine vaccinations through school mandates and aid programs [43,44,45]. Finally, telehealth librarians are able to provide much-needed health education either in rural areas with libraries or remotely through community partnerships.

### Study Limitations

This study was limited to one large southeastern state in the United States. Therefore, the study findings may not be generalizable to other settings with different demographics characteristics, political climate, and attitudes toward vaccines. Furthermore, in the category of school-aged children, the home schooling of children must be considered. Home-schooled children may not have received state-mandated vaccines. Another potential source of inaccuracy relates to the unit of analysis in the de-identified ACHA dataset, which represents inpatient encounters rather than patients. Therefore, individuals being hospitalized multiple times, either for the same or different conditions, may affect population rates. However, given the size of the dataset, the impact is likely minimal and should not significantly affect the overall findings. Any interpretation of the results should be made within the scope of the analysis which focused exclusively on inpatient cases. Therefore, any vaccine-preventable cases treated outside hospitals were not reflected in the data. The data were collected from an administrative database, and thus, the accuracy of the analysis was dependent on the accurate collection of the relevant variables, such as the ICD-10-CM codes used to identify vaccine-preventable cases. Finally, the SVI allowed us, indirectly, to account for the influence of a comprehensive list of socioeconomic variables. Nonetheless, other confounding factors, e.g., individual medical concerns, could also explain decisions to forgo vaccination. We had no way to measure such influences and incorporate them into the model; however, we do not know of any reliable evidence that suggests they would be significant.

## 5. Conclusions

This study highlights the existing gaps in vaccination coverage in a large state in the southeastern part of the United States, particularly in the adult population in rural and socially vulnerable areas. The data underscore the vulnerability of these populations to disease outbreaks, especially in the context of climate instability. Notably, while chickenpox and whooping cough hospitalizations in pre-school-aged children have rebounded to near pre-pandemic levels, pneumococcal-related hospitalizations in this age group have not. This could be potentially due to changes in work settings, daycare use, or the nature of disease transmission. However, this study’s findings are limited to the southeastern region of the country and the findings may not be generalizable to other regions; in addition, the impact of homeschooling vaccinations rates should be considered. The findings of this study support the need for new educational and community-engaged outreach to improve vaccination resources in rural communities, especially those vulnerable to potential natural disasters. Resource allocation to under-served areas in the form of mobile clinics and increases in health information sources leveraging existing resources, such as libraries and telehealth, as well as improved healthcare infrastructure and tailored initiatives, may result in a difference [46].

## Figures and Tables

**Table 1:** Heat map of the number of hospitalizations with an immunizable condition as the principal reason for the episode.

	Pre-School-Aged Children								Children, 5–19 Years of Age								Adults, 20+ Years of Age							
Year	16	17	18	19	20	21	22	23	16	17	18	19	20	21	22	23	16	17	18	19	20	21	22	23
Diphtheria	0	0	0	0	0	0	0	0	0	0	0	0	0	0	0	0	0	0	0	0	0	0	0	0
Poliomyelitis	0	0	0	0	0	0	0	0	0	0	0	0	0	0	0	0	0	0	0	0	0	0	0	0
Measles	0	0	2.6	0.9	0	0	0	0	0	0	0	0	0	0	0	0.3	0	0	0	0	0	0	0	0
Mumps	0	0	1.7	0.9	0	0.9	0.9	0	1.1	1.1	2.0	1.4	0	0	0	0.3	1	0	1	1	0	0	0	1
Rubella	0	0	0	0	0	0	0	0	0	0	0	0	0	0	0	0	0	0	0	0	0	0	0	0
Chickenpox	4.4	7.0	6.1	3.5	0.9	3.7	1.8	6.2	2.0	1.4	2.0	2.0	1.1	0	0.8	1.1	3	4	5	4	3	2	2	2
Hib	0	0	0	0	0	0	0	0	0	0	0	0	0	0	0	0	0	0	0	0	0	0	0	0
Pneumococcal	71	69	131	280	57	44	60	46	19	20	40	68	13	6	12	14	48	55	130	414	92	41	45	47
Hepatitis B	0	0	0	0	0	0	0	0	0	0	0	0	0	0	0	0	0	0	0	0	0	0	0	0
Tetanus	0	0	0	0	0	0	0	0	0	0	0	0	0.3	0	0	0	0	0	0	0	0	0	0	0
Whp-Cough	39	40	34	45	20	0	2	33	0.6	0.3	0.8	1.4	0.3	0	0	2.1	1	1	1	1	1	0	0	1
Hepatitis A	0.9	0.9	1.7	1.8	0	1.8	0.9	1.8	1.4	1.1	3.9	6.4	1.4	2.5	3.5	1.9	24.6	28.6	42.2	126	42.3	15.6	18.5	10.4
HPV High-Risk	0	0	0	0	0	0	0	0	0	0	0	0	0	0	0	0	0.1	0	0.1	0	0	0.1	0.1	0.1
Meningococcal	1.8	1.8	3.5	2.6	1.8	1.8	0.9	0.9	0.6	0.6	0.6	0.3	0	0	0.8	1.1	0.6	0.7	0.8	0.6	0.8	0.5	2.1	0.8
Rotavirus	99	93	59	80	11	23	69	75	8	12	3	6	0	1	7	9	2.2	4.3	2.1	3.3	0.2	0.9	4.3	6.6
RSV	29	11	8	3	0	4	10	5	2	0	1	0	1	0	0	0	0.8	0.4	0.2	0.2	0.1	0.4	0.2	0.2
Shingles	0.9	2.6	1.7	0.9	1.8	2.8	3.6	0.9	4.0	2.0	3.9	2.8	1.6	2.7	1.9	1.6	70	64	68	71	56	53	55	65
COVID-19	0	0	0	0	301	813	1358	674	0	0	0	0	149.7	476	246	77.8	0	0	0	0	4880	8180	3758	1695
Post-COVID-19	0.0	0.0	0.0	0.0	0.0	0.0	0.0	0.0	0.0	0.0	0.0	0.0	0.0	0.8	0.3	0.0	0.0	0.0	0.0	0.0	0.0	0.4	3.4	1.1
Legend		Lowest Value								Medium Value								Highest Value						

**Table 2:** Rates of hospitalizations for immunizable conditions by metropolitan status, race, Hispanic ethnicity, and gender.

	All conditions (Rates/1,000,000)						
Metropolitan Area Status	All	White	Black	Other	Hispanic	Male	Female
	Pre-School-Aged Children						
Large	141.3	108.2	215.5	262.5	69.6	151.8	130.1
Small/Medium	188.0	164.4	207.2	280.8	79.5	188.9	187.7
Micro/Non-Core	225.3	218.1	142.0	449.7	98.8	237.2	205.3
	(*p* < 0.001)	(*p* < 0.01)	(*p* = 0.36)	(*p* = 0.49)	(*p* = 0.81)	(*p* = 0.14)	(*p* = 0.08)
	School-Aged Children						
Large	28.6	23.4	43.1	54.2	10.4	29.2	27.9
Small/Medium	34.0	30.5	49.4	54.1	14.7	36.2	31.7
Micro/Non-Core	62.1	57.9	105.4	31.5	13.6	62.5	60.3
	(*p* = 0.004)	(*p* < 0.001)	(*p* = 0.15)	(*p* = 0.64)	(*p* = 0.43)	(*p* = 0.16)	(*p* = 0.19)
	Adults						
Large	157.8	158.2	119.5	188.9	30.9	163.8	153.7
Small/Medium	182.0	186.7	147.6	191.2	36.1	189.2	176.4
Micro/Non-Core	264.8	279.4	229.9	242.0	28.9	230.2	305.1
	(*p* < 0.01)	(*p* < 0.01)	(*p* = 0.02)	(*p* = 0.68)	(*p* = 0.62)	(*p* = 0.14)	(*p* = 0.01)
	Conditions related to school-mandated vaccines (Rates/1,000,000)						
	Pre-School-Aged Children						
Large	67.0	52.9	88.5	133.1	31.8	78.1	55.5
Small/Medium	88.2	61.7	132.0	167.8	29.5	97.2	79.2
Micro/Non-Core	101.0	77.0	77.3	354.3	50.1	114.1	84.9
	(*p* = 0.31)	(*p* = 0.48)	(*p* = 0.34)	(*p* = 0.26)	(*p* = 0.47)	(*p* = 0.36)	(*p* = 0.18)
	School-Aged Children						
Large	19.6	15.5	35.8	29.8	5.6	20.4	18.7
Small/Medium	26.1	23.1	40.9	38.4	11.4	25.1	27.4
Micro/Non-Core	41.8	38.1	57.2	31.5	8.3	31.2	52.7
	(*p* = 0.02)	(*p* = 0.03)	(*p* = 0.84)	(*p* = 0.77)	(*p* = 0.13)	(*p* = 0.52)	(*p* = 0.05)
	Adults						
Large	106.5	106.3	86.2	123.0	19.1	97.4	116.4
Small/Medium	143.2	146.9	121.4	142.9	28.7	142.1	145.6
Micro/Non-Core	230.6	242.1	215.5	207.7	26.0	192.6	275.5
	(*p* < 0.01)	(*p* < 0.01)	(*p* < 0.01)	(*p* = 0.30)	(*p* = 0.41)	(*p* < 0.01)	(*p* < 0.01)
	Conditions not related to school-mandated vaccines (Rates/1,000,000)						
	Pre-School-Aged Children						
Large	74.3	55.3	127.0	129.4	37.8	73.7	74.6
Small/Medium	99.7	102.7	75.2	113.0	50.0	91.7	108.6
Micro/Non-Core	124.2	141.1	64.7	95.4	48.7	123.1	120.4
	(*p* = 0.32)	(*p* = 0.10)	(*p* = 0.35)	(*p* = 0.89)	(*p* = 0.91)	(*p* = 0.49)	(*p* = 0.65)
	School-Aged Children						
Large	9.0	7.9	7.3	24.4	4.8	8.8	9.2
Small/Medium	7.8	7.4	8.6	15.6	3.3	11.1	4.3
Micro/Non-Core	20.3	19.8	48.1	0.0	5.3	31.3	7.6
	(*p* = 0.02)	(*p* = 0.18)	(*p* = 0.43)	(*p* < 0.01)	(*p* = 0.86)	(*p* = 0.13)	(*p* = 0.66)
	Adults						
Large	51.3	51.9	33.3	65.8	11.8	66.4	37.2
Small/Medium	38.8	39.8	26.2	48.3	7.4	47.1	30.8
Micro/Non-Core	34.2	37.3	14.4	34.3	2.9	37.6	29.6
	(*p* = 0.08)	(*p* = 0.19)	(*p* = 0.04)	(*p* = 0.26)	(*p* < 0.01)	(*p* = 0.01)	(*p* = 0.52)

**Table 3:** Regression results by cohort and age group.

	Adults	School-Aged	Pre-School-Aged
Medium Metropolitan Area	−3.55	1.61	21.28
	(0.704)	(0.433)	(0.042)
Small Metropolitan Area	18.85	3.58	2.50
	(0.103)	(0.159)	(0.846)
Micropolitan Area	3.45	3.77	14.86
	(0.801)	(0.206)	(0.33)
Non-core Area	**26.16**	**7.39**	**23.74**
	**(0.014)**	**(0.005)**	**(0.048)**
SVI	**1.57**	**7.36**	**8.00**
	**(<0.001)**	**(<0.001)**	**(<0.001)**
Year	2.29	0.15	3.46
	(0.171)	(0.694)	(0.065)

Bold values indicate statistical significance at the alpha = 0.05 level.

**Table 4:** Rates of hospitalization with COVID-19 as the principal reason since 2020–2023.

Rate of Inpatient Episodes per 1,000,000 (Pre-School Aged)				
	Large Metropolitan Area	Small/Medium Metropolitan Area	Micro/Non-Core Metropolitan Area	(*p*-Value)
All groups	344	403	460	<0.001
White	266	323	397	<0.001
Black	391	467	515	<0.001
Other NWB	790	918	1004	<0.001
Hispanic	188	239	161	<0.001
Male	313	333	413	<0.001
Female	374	470	505	<0.001
Rate of Inpatient Episodes per 1,000,000 (School-Aged)				
	Large Metropolitan Area	Small/Medium Metropolitan Area	Micro/Non-Core Metropolitan Area	(*p*-value)
All groups	120	110	145	0.002
White	85	87	118	0.003
Black	170	152	200	<0.001
Other NWB	260	220	339	<0.001
Hispanic	59	64	88	0.007
Male	118	109	162	<0.001
Female	122	110	129	0.093
Rate of Inpatient Episodes per 1,000,000 (Adults)				
	Large Metropolitan Area	Small/Medium Metropolitan Area	Micro/Non-Core Metropolitan Area	(*p*-value)
All groups	2310	2209	2513	<0.001
White	2044	2062	2333	<0.001
Black	2807	2717	2545	0.001
Other NWB	4319	3745	6930	<0.001
Hispanic	1192	993	963	0.001
Male	2192	2050	2659	<0.001
Female	2437	2378	2386	0.473

## Data Availability

The original contributions presented in this study are included in the article. Further inquiries can be directed to the corresponding author.
